# Shared variance in creative self-beliefs: examining self-compassion and personality traits in creative adolescents and adults

**DOI:** 10.3389/fpsyg.2026.1812481

**Published:** 2026-05-04

**Authors:** Michael P. Diana, Haiying Long, Barbara A. Kerr, Randi C. Gray, Taylor P. Harris

**Affiliations:** Department of Educational Psychology, University of Kansas, Lawrence, KS, United States

**Keywords:** creative personality, creative self-beliefs, creativity, openness to experience, self-compassion

## Abstract

**Introduction:**

Creative self-beliefs are shaped by stable personality traits and psychological resources. While the links between the creative personality and creative self-beliefs are established, the role of self-compassion, a promoter of subjective well-being, in shaping creative self-beliefs remains a significant theoretical gap. The goal of this study was to describe the relationships among creative self-beliefs, personality traits, and self-compassion in a sample of creative adolescents and adults.

**Methods:**

Creative adolescents and adults were recruited using a purposive sampling procedure in the Midwestern United States. Participants were recruited from a creative career counseling workshop where their creative status was verified through a teacher’s nomination and/or creative accomplishments. A cross-sectional sample of 127 creative adolescents and adults completed self-report measures assessing personality, self-compassion, and creative self-beliefs. Hierarchical linear regression modeled the predictive relationships of these variables, with age group included as a categorical, control variable to account for developmental differences.

**Results:**

Correlations showed positive relationships between creative self-beliefs and self-compassion. Hierarchical regressions revealed that self-compassion significantly predicted creative self-beliefs in the initial model. Once personality characteristics were included, openness to experience and conscientiousness emerged as the strongest predictors. The results indicate that while self-compassion is associated with creative self-beliefs, it does not account for unique variance beyond that explained by core personality traits.

**Discussion:**

These findings suggest that self-compassion may promote creative self-beliefs by encouraging positive self-perceptions. Although the predictive power of self-compassion overlaps with broader personality traits, identifying both stable and malleable contributors to creativity offers clear targets for psychological and educational interventions across the lifespan. Limitations, implications, and future research directions are discussed.

## 1 Introduction

Various psychological factors, including personality, self-beliefs, and emotional well-being, are critical in shaping an individual’s capacity to generate novel ideas and solve problems. The creative personality encompasses traits such as openness to experience, providing the ideational and motivational foundation for creative output (Kaufman, 2013). It may be paired with creative self-beliefs, the confidence in one’s creative abilities (Karwowski and Barbot, 2016). Self-compassion, which involves treating oneself with kindness during moments of failure or criticism (Neff, 2003), has been linked to enhanced creativity by buffering the effects of self-judgment (Zabelina and Robinson, 2010). Research is emerging on relationships between creative constructs and self-compassion; however, the precise relationships among self-compassion, creative personality, and creative self-beliefs remain a significantly underexplored area in creativity research.

Creative self-beliefs have gained increased empirical attention with research examining personality traits (Karwowski and Lebuda, 2016; Pavlić et al., 2023) and cognitive components (Karwowski and Barbot, 2016). These trait-based and cognitive models account for the substantial variance observed in creativity (Puryear et al., 2017); however, not all variance is accounted for. Individuals with similar personality traits may differ in creative self-perceptions and motivation, which may be accounted for by differences in emotional capabilities (Ivcevic and Brackett, 2015). Therefore, understanding how social–emotional and cognitive-evaluative resources, such as self-compassion, relate to creative self-beliefs is essential. Self-compassion represents a multidimensional process comprising mindfulness, common humanity, and self-kindness (Neff, 2003) which may support the self-referential nature of creativity.

The contemporary relevance of investigating creative self-beliefs, personality, and self-compassion is defined by the need for sustained creative motivation and persistence across the lifespan. While individual creative self-efficacy is often viewed through the lens of cognitive ability, the ability to maintain positive self-perceptions through the ambiguity and setbacks inherent in creative work is equally vital (Beghetto and Karwowski, 2023). Examining how self-compassion and personality relate to creative self-concept can clarify the factors that support creative persistence and perception of creative abilities. Understanding these relationships is critical, as creative self-beliefs may motivate individuals to persist through the challenges encountered in academic environments (Beghetto, 2006), occupational settings (Gong et al., 2009), and professional development (Tierney and Farmer, 2011).

Creativity research discusses the paradoxical nature of creative individuals as they are characterized by internal conflict, emotional volatility, and an increased vulnerability to psychopathology (Simonton, 2012). While emotional instability may impact creativity, it becomes essential to understand how emotional resources, like self-compassion, can be used to mitigate the effects of negative affectivity (Poka et al., 2024). The way self-compassion interacts with personality may serve as a motivator to persist through creative challenges encountered at the individual level. A critical gap in existing creativity models is the failure to address how self-compassion, a potential promoter of creative self-beliefs, may strengthen or weaken the link between an individual’s personality and their perceptions of their creative abilities.

## 2 Literature review

### 2.1 Creative self-beliefs

Creative self-beliefs are an individual’s definition of creativity and their overall thoughts and expectancies regarding their own creative potential and abilities (Karwowski and Barbot, 2016). Creative self-beliefs encompass creative awareness, creative self-efficacy, and creative self-image (Karwowski et al., 2019a,b). Creative self-efficacy, the self-perceptions of an individual’s abilities to create novel solutions to solve problems requiring creativity (Beghetto, 2006), serves as a foundational component of creative self-beliefs (Karwowski, 2016). Creative self-beliefs represent the broader self-referential framework through which individuals perceive their creative abilities.

Empirical studies consistently demonstrate that individuals with stronger creative self-beliefs are more likely to engage in divergent thinking, exhibit higher creative performance, and pursue creative goals with greater persistence (Tierney and Farmer, 2002; Pretz and Nelson, 2017). Researchers emphasize that creative self-beliefs are not static; while beliefs show short-term stability, they are a dynamic process that varies across the lifespan (Karwowski, 2016). For instance, significant changes are often noted within the late adolescence to emerging adulthood, highlighting the need to account for developmental factors when studying creative self-beliefs (Karwowski, 2016). Cross-cultural research indicates that creative self-beliefs mediate the relationship between cultural values and creative expression, highlighting that the role of social norms may impact beliefs about creativity (Tang et al., 2018). Additionally, studies have linked higher creative self-efficacy and creative personal identity to greater psychological well-being and adaptive coping, suggesting that creative self-beliefs extend beyond creativity to influence overall well-being (Puente-Díaz and Cavazos-Arroyo, 2017).

Creative self-beliefs are shaped by personality and contextual factors. Among personality traits, openness to experience consistently emerges as the strongest predictor of creative self-beliefs (Karwowski and Lebuda, 2016; Silvia et al., 2009). Conversely, neuroticism has been negatively associated with creative self-beliefs (Feist, 1998; Karwowski et al., 2013; Pavlić et al., 2023), potentially due to heightened self-doubt and sensitivity to evaluation. Beyond openness and neuroticism, traits such as conscientiousness and extraversion have shown positive, albeit weaker, associations with creative self-efficacy, possibly reflecting the motivational and social components of creative engagement (Karwowski and Lebuda, 2016). Research has shown that mastery experiences, autonomy-supportive environments, and exposure to creative role models enhance creative self-efficacy and, in turn, creative engagement (Karwowski, 2014). Conversely, environments that emphasize evaluation, conformity, or rigid performance standards can undermine creative self-beliefs by heightening self-doubt and fear of negative judgment (Beghetto and Dilley, 2016).

### 2.2 Creative personality

The creative personality is a stable pattern of traits that provide the motivation and behaviors necessary for an individual to engage in a process that yields a product that is both novel and useful as defined within a domain (Kaufman and Sternberg, 2010). The most consistent and robust component of the creative personality profile is openness to experience (Feist, 1998; Puryear et al., 2017). This trait, which encompasses an individual’s propensity toward fantasy, aesthetics, imagination, and unconventional ideas and values, is widely recognized as the major positive predictor of both creative self-perceptions and achievement (Feist, 1998; Da Costa et al., 2015; Grajzel et al., 2023; Kaufman et al., 2016). Openness to experience is believed to promote intelligence (Kandler et al., 2016) and cognitive flexibility (Chen et al., 2022) which are foundational to divergent thinking. Traits related to openness to experience (e.g., curiosity, risk-taking, and non-conformity) are key aspects of the creative personality (Selby et al., 2005). Creative individuals have consistently been shown to perform well on intelligence tests, indicating the high cognitive capacity necessary for complex problem-solving (Kandler et al., 2016). While openness drives the disposition to create, the process of bringing those ideas to fruition often involves other traits, such as conscientiousness, which contributes to the necessary discipline and perseverance.

Extraversion and novelty seeking have been consistently described as a positive correlate to the creative personality and are believed to assist with assertiveness and social engagement necessary to promote creative works (Gocłowska et al., 2019). The interaction of emotional traits, states, and arousal can impact creative performance (Zenasni and Lubart, 2008) as emotional intelligence has been demonstrated to correlate positively with creative performance (Wolfradt et al., 2002). Meta-analyses have supported the evidence that traits like openness to experience and extraversion are strong positive predictors of creativity (Feist, 1998; Grajzel et al., 2023; Puryear et al., 2017), establishing these as traits consistent with this personality profile.

While high openness to experience and extraversion are consistently positively correlated with creativity (Puryear et al., 2017), research also denotes a strong negative correlation between creativity and neuroticism (Pickering et al., 2016). However, it is believed that greater neuroticism may increase creative performance and potential under the right circumstances (Baas et al., 2013). In the most recent decade, creative students reported higher levels of anxiety, depression, and suicidality than previously, indicating that neuroticism may negatively impact expression (Kerr et al., 2021). The negative association suggests that emotional instability may inhibit the psychological processes necessary for creative and innovative thinking.

Measures of the Big Five personality traits are common instruments used to assess creative personality. Developed by McCrae and Costa (1999), this model provides a comprehensive framework for personality structured around five main domains: neuroticism, extraversion, openness to experience, agreeableness, and conscientiousness. This instrument is used in both research and clinical settings, providing valuable insights into personality traits and their correlations with various outcomes, such as mental health, interpersonal relationships, and creativity. Research into creative personalities, such as work by Kerr and McKay (2013) using the NEO Personality Inventory-3 (NEO-PI-3) with creative adolescents, has further affirmed the relevance of the Big Five personality traits and creativity.

While understanding the individual traits of the creative personality is important, researchers have categorized the Big Five factors into two higher-order meta-traits: plasticity and stability. Plasticity is comprised of openness to experience and extraversion while stability is made up of conscientiousness, agreeableness, and neuroticism (Feist, 2019; Silvia et al., 2009). Plasticity promotes engagement with novel stimuli and tolerance of ambiguity (Silvia et al., 2009) and stability provides a self-regulatory framework for creating and editing products (Feist, 2019). Plasticity encourages behavioral and cognitive change in response to environmental shifts with stability ensuring the goal-directed persistence that is needed to bring ideas to fruition (Stamps and Biro, 2016). Ultimately, the creative self-concept relies on the interaction of these meta-traits, where plasticity enables the exploration and stability buffers the individual against setbacks encountered in the creative process (Feist, 2019; Stamps and Biro, 2016).

### 2.3 Self-compassion

Self-compassion is a non-judgmental stance toward one’s suffering, involving a balanced recognition of painful experiences and responding kindly rather than self-criticism (Neff, 2003). Self-compassion is composed of three oppositional components: self-kindness versus self-criticism, common humanity versus isolation, and mindfulness versus overidentification. Self-kindness rejects criticism by embracing grace; common humanity situates that suffering is universal, while mindfulness promotes acceptance of positive and negative emotions (Neff, 2003).

Research in the last decade has associated self-compassion with creativity (Walton et al., 2025; Verger et al., 2022; Zabelina and Robinson, 2010). Self-kindness replaces the harsh, judgmental inner voice with a supportive one, which is necessary, as creativity often involves perceived failure and rejection (Manalo and Kapur, 2018). A self-critical stance can negatively impact idea generation and further prevent risk-taking, while self-kindness buffers against judgmental self-perceptions (Zabelina and Robinson, 2010). Common humanity counters the feeling of isolation by acknowledging that imperfection and struggle are universal to creativity. It can remind individuals that creative blocks and critical feedback are universal experiences, thereby potentially reducing shame often associated with creative setbacks (Ye et al., 2025). Mindfulness has been positively linked to creativity (Henriksen et al., 2020) as it appears to promote a balanced acknowledgement of creative accomplishments and challenges. This balanced perspective remains consistent with the conceptualization of mindfulness within a self-compassion framework (Neff, 2003). These components promote resilience and emotional regulation (Neff and Germer, 2013), skills which may impact creative self-perceptions.

Historically, self-compassion was studied in mental health (MacBeth and Gumley, 2012), academics (Neff et al., 2005), and general healthcare sectors (Raab, 2014). More recently, empirical evidence supports that self-compassion is associated with increased creative originality, particularly among self-judgmental individuals, because self-compassion appears to buffer negative self-talk (Zabelina and Robinson, 2010). Self-compassion mediates emotional dysregulation and creative achievement, serving as a potential protective factor facilitating creative exploration (Verger et al., 2022). Additionally, recent research indicates that self-compassion interventions lead to better mental health of performing artists (Walton et al., 2025) and this relationship may generalize to other creative professions. While research on self-compassion and general creativity (Glossop, 2014), performing arts (Walton et al., 2025), verbal creativity (Bellosta-Batalla et al., 2021) and creative originality (Zabelina and Robinson, 2010) are promising, there is necessity for research examining how self-compassion interacts with more specific constructs, such as creative self-beliefs.

### 2.4 Current study

The current study investigates the relationships among personality characteristics, self-compassion, and creative self-beliefs with a sample of creative adolescents and adults. Creativity is inherently a multidimensional construct, influenced significantly by an individual’s stable personality traits, self-perceptions, and emotional well-being. Studying these interconnected factors is essential for a nuanced and holistic understanding of creativity. Existing literature provides a strong foundation, consistently linking personality traits like openness to experience and creative self-beliefs (Karwowski and Lebuda, 2016) and personality traits and creative self-concept (Gupta et al., 2024; Karwowski and Lebuda, 2017). Furthermore, research demonstrates that self-compassion is related to increased creative output, particularly among individuals prone to self-judgment (Zabelina and Robinson, 2010).

The study examines self-compassion as a psychological resource associated with creative self-beliefs, potentially accounting for variance not captured by stable personality traits alone. This potential promotive role may allow individuals to protect their creative self-perceptions during the inevitable failure involved in creative tasks. However, before the nature of this complex interaction can be established, the current body of research is unclear regarding the precise way self-compassion interacts with both the creative personality and creative self-beliefs simultaneously.

The current study aims to answer the overarching research question, “What are the relationships among personality characteristics, creative self-beliefs, and self-compassion in creative adolescents and adults?” Given that, to our knowledge, this is the first attempt to examine this specific three-way relationship, the study is focused on establishing the unique variance contributed by personality and self-compassion on creative self-beliefs while accounting for developmental differences between adolescent and adult participants.

## 3 Methods

### 3.1 Participants

Participants were recruited through the Counseling Laboratory for the Exploration of Optimal States (CLEOS) project, a career counseling workshop designed for creative individuals at a large Midwestern university in the United States. The CLEOS project assists creative adolescents and adults by providing personality and creativity assessments to understand their accomplishments, pursuits, and career interests (Kerr and McKay, 2013). While the project initially focused on adolescent career pathways, its recent expansion to include adult participants provides a valuable opportunity to investigate creative development across the lifespan.

The CLEOS project recruited creative individuals between 2006 and 2024 with a total of 1,263 participants throughout the 18-year tenure of the project. A total of 133 participants were invited to participate and completed the CLEOS career counseling workshop between January 2023 and May 2024. The analysis focuses specifically on these cohorts, as they were the only groups to complete the Self-Compassion Scale-Short Form (SCS-SF; Raes et al., 2011). Six participants were excluded due to incomplete responses on the SCS-SF, resulting in a final sample of 127 participants.

The study used a two-fold purposive sampling strategy: a criterion-based nomination for adolescents and self-selection with credential verification for adults. Participants were recruited from the Midwestern United States using a targeted purposive sampling method for adolescents and adults. Adolescent participants were recruited from multiple high schools across two Midwestern states. Teachers identified adolescents based on profiles that included creative behaviors, demonstrated creative accomplishments (e.g., awards, portfolio work), and expressed interest in creative careers. Teachers were provided with a broad definition of creativity to ensure diverse representation of students. Adult participants were recruited through advertisements within an honors program at a large Midwestern university, as well as through social media posts and email campaigns targeting creative communities. Adult participants were self-selected into the program. However, all adult participants provided a list of their creative accomplishments to qualify for inclusion, ensuring that the sample was composed of creative individuals.

The final analytic sample (*N* = 127) had a mean age of 20.76 (ranging from 15 to 57, *SD* = 7.23). Participants identified as female (52%), male (44%), and nonbinary (4%). Regarding race and ethnicity, participants identified as White (69%), multiracial (12%), Hispanic (6%), African American (5%), Asian American (3%), and did not disclose race (4%).

While the primary analysis used age groups to mirror the recruitment structure of the CLEOS project, supplemental analyses were conducted treating age as a continuous variable. Because developmental research suggests specific shifts in creative self-beliefs between adolescence and adulthood (Karwowski, 2016), the categorical approach was retained to ensure the model accounted for these distinct cohorts. In our dataset, we characterized participants between ages 14–19 as adolescents and participants aged 20 and above as adults.

To determine the statistical power, a sensitivity analysis was conducted using G*Power 3.1 to determine the minimum detectable effect size for the hierarchical regression model (6 predictors, *α* = 0.05, *N* = 127). The analysis indicated the study was sufficiently powered (1 − *β* = 0.80) to detect a Cohen’s *f^2^* of 0.11. Because the current investigation uses an existing dataset with a final sample size of *N* = 127, the sample was deemed sufficiently powered to investigate the relationships among personality characteristics, self-compassion, and creative self-beliefs.

### 3.2 Measures

#### 3.2.1 Creative self-beliefs

Creative self-beliefs were measured using the Short Scale of the Creative Self (SSCS; Karwowski et al., 2018). The SSCS consists of 11 items and can be answered on a 5-point or a 7-point Likert scale with both the 5-point and 7-point scales having adequate reliability (Zielińska et al., 2022). This study used the 5-point Likert Scale ranging from “Definitely Not” (1) to “Definitely Yes” (5). The SSCS has two subscales: Creative Personal Identity; and Creative Self-Efficacy. Creative Personal Identity items assess a participant’s perception of the importance of creativity as part of themselves (e.g., “I think I am a creative person”). Creative Self-Efficacy items measure a participant’s beliefs about their abilities to solve problems using creativity (e.g., “I trust my creative abilities”). Responses from these two subscales can be added together to create a composite score known as Creative Self-Concept. The researchers used the Creative Self-Concept composite score because it is applicable and comparable across the diverse age groups in the sample (Zielińska et al., 2022). The SSCS demonstrates discriminant and convergent validity because scores are positively related to creative ability, intrinsic motivation, self-esteem, and self-rated originality (Karwowski et al., 2018). Cronbach’s alpha for the SSCS in the current study was 0.86, demonstrating good reliability. SSCS takes approximately 5 min to complete, and participants completed this as a remote, self-paced digital assessment via Qualtrics approximately 24 h prior to their workshop attendance.

#### 3.2.2 Personality

Personality characteristics were measured using the NEO-PI-3 (McCrae et al., 2005), a personality assessment based on the five-factor model of personality, which includes the Big Five personality traits: openness to experience, conscientiousness, extraversion, agreeableness, and neuroticism. The NEO-PI-3 has 240 items on a 5-point Likert scale that ranges from “Strongly Disagree” (1) to “Strongly Agree” (5). Each factor is measured by 48 items (6 facets x 8 items per facet), with the final factor score calculated as the sum of its items (McCrae et al., 2005). Each of the five factors is composed of six facets (totaling 30 facets) to provide a more nuanced explanation of personality. Cronbach’s alpha for the full scale is 0.75 in the current study, demonstrating adequate internal reliability. The NEO-PI-3 typically takes 30–45 min to complete, and participants completed this as a remote, self-paced digital assessment via Qualtrics approximately 24 h prior to their workshop attendance.

#### 3.2.3 Self-compassion

Self-compassion was measured by the Self-Compassion Scale-Short Form (SCS-SF; Raes et al., 2011) that contains 12-items on a 5-point Likert scale from “Almost Never” (1) to “Almost Always” (5). The scale assesses how often participants engage in self-compassionate thoughts and behaviors. There are no clinical norms because scores are used on an individual level to indicate higher or lower levels in self-compassion (Raes et al., 2011). High scorers in self-compassion range from 3.5 to 5, with average scores between 2.6 and 3.4, and low scorers between 0 and 2.5 (Raes et al., 2011). SCS-SF has six subscales that assess participants’ self-kindness, common humanity, mindfulness, self-criticism, isolation, and overidentification. SCS-SF contains 2 items from each of the subscales of the original long-form Self-Compassion Scale (SCS; Neff, 2003). SCS-SF has a strong correlation with the long-form SCS (*r* = 0.97) and demonstrates adequate internal consistency (Cronbach’s alpha = 0.86, Raes et al., 2011). SCS-SF shows a higher-order self-compassion factor and six two-item factors. Raes et al. (2011) caution researchers when using the individual subscales because each subscale only contains two-items with reliability ranging from 0.54–0.75. For the current study, Cronbach’s Alphas for the subscales ranged between 0.53–0.81. Given the limited item count per subscale (2 items each) and the resulting variability in reliability, subscale-level analyses were omitted in favor of the more robust composite self-compassion score. The overall self-compassion composite score was calculated by first reverse-scoring the six negatively worded items (self-criticism, isolation, and overidentification) and then averaging all 12 items. Cronbach’s alpha of this measure in the current study is 0.82, demonstrating good reliability. SCS-SF takes approximately 5 min to complete, and participants completed this as a remote, self-paced digital assessment via Qualtrics approximately 24 h prior to their workshop attendance.

### 3.3 Procedure

This study (IRB # STUDY00141951) was approved by an Institutional Review Board at a large Midwestern university in the United States with continuing reviews being approved since the first approval. Recruitment for this study took place from January 2023–May 2024.

The researchers used an IRB-approved recruitment letter and sent information to high schools in two Midwestern states, a large Midwestern university, contacted creative communities over email, and posted on social media looking for interested participants. Adolescent participants were nominated by teachers based on their creative accomplishments and interests in creative careers. Once adolescents were nominated, the researchers sent a consent form to the teacher and the school with the study information for parents to consent for their child to participate in the study. If given consent, the caregivers provided demographic information about their child (including email) so researchers could send data collection measures.

Adult participants self-selected into this study by contacting the lead researcher of the CLEOS project or a research assistant via email. During initial screening, potential adult participants provided the researchers with a list of creative accomplishments. If participants met screening criteria, adult participants were provided with a detailed informed consent form outlining the study’s purpose and confidentiality, which was obtained digitally prior to starting the survey.

Using the emails provided by participants and/or their caregivers, the researchers emailed participants with a secure link to complete the survey measures hosted on Qualtrics. The Qualtrics survey included the measures relevant to this study (personality, self-compassion, and creative self-beliefs), alongside demographic questions, career interest inventories, and other creativity assessments. Participants attended the career counseling workshop in-person or virtually between January 2023–May 2024 and met with counselors-in-training to review assessment results with this study being a retrospective analysis of the data collected.

### 3.4 Data analysis

All statistical analyses were conducted using IBM SPSS Statistics (Version 29). Prior to the main hierarchical regression analyses, a Missing Value Analysis (MVA) using Little’s Missing Completely at Random (MCAR) test indicated that missing data was completely at random, *χ*^2^ *(13)* = 10.21, *p* = 0.63. Levene’s Test was not significant, *F*(1, 125) = 1.18, *p* = 0.28, indicating that the assumption of homogeneity of variance was met between the adolescent and adult participants, justifying inclusion in a single model.

Before conducting the hierarchical linear regression, all models were thoroughly assessed for violations of statistical assumptions, including multicollinearity, normality, linearity, independence, and homoscedasticity, and no major violations of assumptions were detected. Given the theoretical and empirical overlap between self-compassion and neuroticism, multicollinearity was assessed using Variance Inflation Factors (VIF) and tolerance statistics. All VIF values in the final model were within acceptable limits (all VIF < 1.80), with self-compassion and neuroticism both yielding a VIF of 1.790 and a tolerance of 0.559. Other predictors, including openness to experience (VIF = 1.338) and conscientiousness (VIF = 1.064), also demonstrated low collinearity. These results confirm that while self-compassion and personality share significant variance, they are statistically distinct predictors that do not bias the regression estimates.

The analysis proceeded in two primary stages on the final sample (*N* = 127). First, descriptive statistics and bivariate correlation analyses were computed to examine means, standard deviations, and relationships among the Big Five personality factors (neuroticism, extraversion, openness, agreeableness, conscientiousness), self-compassion, and creative self-beliefs. Second, to address the research questions, a hierarchical linear regression analysis was conducted with creative self-beliefs serving as the dependent variable. The predictor variables were entered into SPSS in three sequential steps: Step 1 included only self-compassion; Step 2 added the Big Five personality factors; and Step 3 added age group as a categorical, control variable to account for developmental differences as our sample contained adolescents and adults.

To address potential common method variance, a post-hoc Harman’s Single-Factor Test indicated that the first factor accounted for only 19.10% of the total variance, which is significantly below the 50% threshold commonly used to indicate pervasive common method bias (Podsakoff et al., 2003).

## 4 Results

Descriptive statistics and bivariate correlations among study variables were calculated for the full sample (*N* = 127) and are presented in Table 1. On average, participants showed a moderate level of self-compassion (*M* = 2.84, SD = 0.58), while they showed high creative self-beliefs (*M* = 44.08, SD = 6.85). Regarding personality, participants scored highest on openness to experience (*M* = 61.49, SD = 12.26) and lowest on extraversion (*M* = 44.17, SD = 12.63).

**Table 1:** Descriptive statistics and correlations.

	M	SD	SC	CSB	OPEN	EXT	NEUR	AGR	CON
SC	2.84	0.58	–						
CSB	44.08	6.85	0.18*	–					
OPE	61.49	12.26	0.15	0.42**	–				
EXT	44.17	12.63	0.14	0.24**	0.16*	–			
NEU	57.66	11.45	−0.59**	−0.097	0.19*	−0.11	–		
AGR	54.43	12.17	−0.06	−0.087	0.37**	−0.05	0.15*	–	
CON	52.35	12.30	0.15	0.21*	−0.045	0.015	−0.011	−0.13	–

*p < 0.05; **p < 0.001; #marginally significant.

To address potential developmental differences between adolescent and adult participants, descriptive statistics and independent samples t-tests were performed. Adolescent participants (*N* = 57) showed a moderate level of self-compassion (*M* = 2.78, SD = 0.59) and high creative self-beliefs (*M* = 43.14, SD = 7.35). For personality characteristics, adults scored highest on openness (M = 56.92, SD = 9.94), with neuroticism being a close second (*M* = 55.56, SD = 11.18) and extraversion as the lowest (*M* = 40.96, SD = 11.75). Adult participants (*N* = 70) showed a moderate level of self-compassion (*M* = 2.87, SD = 0.58) and high creative self-beliefs (*M* = 44.84, SD = 6.36). For personality characteristics, adolescents scored highest on openness (M = 65.99, SD = 12.19), with extraversion as the lowest (*M* = 46.79, SD = 12.79).

Independent samples t-tests were conducted to compare mean scores between the adolescent and adult cohorts. Levene’s Test indicated that equal variances were assumed across all variables with each statistic being greater than 0.05. Results indicated no significant differences between adolescent and adult participants on creative self-beliefs, self-compassion, agreeableness, and neuroticism. Neuroticism approached significance (*p* = 0.06), indicating that more power may reveal a significant difference between adults and adolescents. Adults reported significantly higher levels of openness to experience [*t*(127) = −5.11, *p* < 0.001], extraversion [*t*(127) = −2.88, *p* = 0.005], and conscientiousness [*t*(127) = −2.24, *p* = 0.027] when compared to adolescents.

For the full sample, Bivariate Pearson correlations revealed that self-compassion was significantly and positively correlated with creative self-beliefs (*r* = 0.18, *p =* 0.02). Self-compassion was significantly and negatively associated with neuroticism (*r* = −0.59, *p <* 0.001). Correlations between self-compassion and openness to experience (*r* = 0.15, *p* = 0.05) and self-compassion and conscientiousness (*r* = 0.15, *p* = 0.05) were marginally significant. There were no significant correlations between self-compassion and extraversion or agreeableness.

Creative self-beliefs demonstrated significant positive correlations with three personality factors: openness to experience (*r* = 0.42, *p <* 0.001), extraversion (*r* = 0.24, *p* = 0.004), and conscientiousness (*r* = 0.21, *p =* 0.008) but were not significantly related with either neuroticism or agreeableness. Table 1 presents all Bivariate Pearson Correlations.

A hierarchical linear regression analysis was conducted to examine whether self-compassion, the Big Five personality traits, predicted creative self-beliefs after adding age as a categorical, control variable (see Table 2). In Step 1 of the regression model, self-compassion was entered as the lone predictor of creative self-beliefs. The model was significant, *F*(1, 125) = 4.01, *p* = 0.047, only accounting for 3.1% of the variation in creative self-beliefs. Self-compassion was found to be a significant, positive predictor of creative self-beliefs (*β* = 0.18, *p* = 0.047).

**Table 2:** Hierarchical linear regression.

Predictor	Step 1					Step 2					Step 3				
	*β*	*b*	SE	*t*	*p*	*β*	*b*	SE	*t*	*p*	*β*	*b*	SE	*t*	*p*
(Constant)		38.22	0.04	12.80	<0.001**		27.20	6.32	4.30	<0.001**		25.87	6.34	4.08	<0.001**
SC	0.18	2.07	1.03	2.00	0.047*	−0.06	−0.74	1.21	−0.61	0.54	−0.05	−0.62	1.20	−0.52	0.61
NEU	–	–	–	–	–	−0.20	−0.12	0.06	−1.95	0.05#	−0.18	−0.11	0.06	−1.73	0.09
OPE	–	–	–	–	–	0.46	0.26	0.05	5.20	<0.001**	0.51	0.28	0.05	5.45	<0.001**
EXT	–	–	–	–	–	0.15	0.08	0.04	1.83	0.07	0.17	0.09	0.04	2.12	0.04*
AGR	–	–	–	–	–	−0.01	−0.01	0.05	−0.15	0.88	−0.01	−0.004	0.05	−0.08	0.94
CON	–	–	–	–	–	0.24	0.13	0.04	2.99	0.003*	0.27	0.15	0.05	3.28	0.001*
AGE	–	–	–	–	–	–	–	–	–	–	−0.14	−1.90	1.22	−1.56	0.12

**p < 0.001; *p < 0.05; #marginally significant.

Step 2 of the model included the Big Five personality characteristics in addition to self-compassion. The overall model was significant, *F*(6, 120) = 8.12, *p* < 0.001, accounting for 28.9% of the variance in creative self-beliefs, indicating a significant increase in variance accounted for from personality variables. The addition of the Big Five personality factors in Step 2 resulted in a significant increase in explained variance, ΔR^2^ = 0.26, *F* change(5, 120) = 8.71, *p* < 0.001. Openness to experience (*p* < 0.001) and conscientiousness (*p* = 0.003) were both significant, positive predictors of creative self-beliefs, with openness to experience showing a stronger effect (*β* = 0.46) than conscientiousness (*β* = 0.24). The effects of neuroticism and extraversion were marginally significant (*p* = 0.05; 0.07, respectively), with neuroticism being a negative predictor (*β* = −0.20) and extraversion (*β* = 0.15) being a positive predictor. The effect of agreeableness was not significant (*β* = −0.01, *p* = 0.88).

After adding the Big Five personality variables, self-compassion (*β* = −0.06, *p* = 0.54) was not a significant predictor with the direction of the relationship shifting from positive to negative. This shift suggests that the initial association between self-compassion and creative self-beliefs was largely accounted for by the shared variance between self-compassion and the Big Five factors, particularly neuroticism. The change in the self-compassion coefficient upon the entry of the Big Five factors indicates a redundancy effect, where the initial predictive power of self-compassion was subsumed by its shared variance.

Step 3 of the model added age group as a categorical, control variable in addition to self-compassion and personality. The overall model was significant, *F*(1, 119) = 7.39, *p* < 0.001, *R^2^* = 0.30, showing a slight increase in the variance explained by group (30% compared to 28.9%). The addition of age group as a categorical control variable in Step 3 resulted in a non-significant increase in explained variance, ΔR^2^ = 0.011, *F* change (7, 119) = 2.45, *p* = 0.12. Categorical age was not a significant predictor of creative self-beliefs (*β* = −0.14, *p* = 0.12), suggesting that participants, regardless of age, had comparable creative self-beliefs. Although self-compassion was still not significant (*β* = −0.05, *p* = 0.61), openness to experience (*β* = 0.51, *p* < 0.001) and conscientiousness (*β* = 0.27, *p* = 0.001) remained the strongest positive predictors of creative self-beliefs. Extraversion became a significant, positive predictor (*β* = 0.17, *p* < 0.05). Neuroticism’s effect was not significant (*β* = −0.18, *p* = 0.09), and agreeableness remained nonsignificant (*β* = −0.01, *p* = 0.94). These results indicate that while self-compassion is associated with creative self-beliefs, its predictive utility does not extend beyond the Big Five personality traits, which remain the primary contributors to the variance.

## 5 Discussion

The purpose of the study was to describe the relationships among self-compassion, creative self-beliefs, and personality variables in a sample of creative adolescents and adults. Prior research investigating personality and self-concept to creativity (Gupta et al., 2024; Karwowski and Lebuda, 2017) served as the theoretical basis for this study. Specifically, this study aimed to examine the predictive relationships among self-compassion, personality traits, and creative self-beliefs. Acknowledging that we had adolescent and adult participants, participants were categorized by age and added to the regression model as a categorical, control variable to determine if age impacted results. The rationale for categorizing age is reflected by Karwowski’s (2016) study demonstrating that creative self-beliefs shifted between late adolescence and early adulthood. By accounting for developmental differences between adolescents and adults, this study provides a nuanced look at how stable personality traits and malleable emotional processes impact creative self-beliefs.

While the current study included both adolescent and adult participants, it is important to clarify that the primary objective was not comparative developmental analysis, but rather an investigation into the overarching predictive relationships among self-compassion, personality traits, and creative self-beliefs. The inclusion of two distinct age groups was intended to increase the diversity of the sample, allowing for a more nuanced understanding of the primary constructs. By treating age group as a categorical control variable in the hierarchical regression, the study aimed to determine if the predictive power of self-compassion and personality remained consistent regardless of developmental stage. This approach prioritizes the identification of potential interactions among variables instead of a cross-sectional comparison. Although the goal was not to compare directly, independent samples t-tests were conducted to assess mean differences.

The lack of significant differences between adolescents and adults regarding creative self-beliefs (*p* = 0.25) and self-compassion (*p* = 0.61) suggests potential stability in these internal processes. This is consistent with previous research suggesting that while creative performance may fluctuate, creative self-beliefs may stabilize in emerging adulthood (Karwowski, 2016). However, significant differences emerged across several personality domains, with adults reporting higher levels of openness to experience (*p* < 0.001), extraversion (*p* = 0.005), and conscientiousness (*p* = 0.027). Plasticity (openness and extraversion) being two of the significant differences reflects a strong connection to the creative personality (Feist, 2019). Findings suggest that while adults may possess a more robust creative personality profile (Feist, 2019), their actual confidence in their creative abilities appears to not differ from that of younger participants. Despite the personality gaps, findings reinforce the idea that creative self-perceptions may be filtered through a complex set of fixed and malleable appraisals (Karwowski et al., 2019b).

Self-compassion’s positive and significant association with creative self-beliefs establishes a theoretical link between the constructs. Self-compassion was not significantly correlated to openness or conscientiousness, which were the strongest predictors of creative self-beliefs. Though not significant, the correlations of openness, extraversion, and self-compassion were positive, which is consistent with recent research highlighting self-compassion and openness’s positive relationship (Sohail and Bukhari, 2025). Neuroticism was strongly and negatively correlated to both self-compassion and creative self-beliefs, which is consistent with prior work showing that high neuroticism is associated with lower creative self-beliefs (Karwowski and Lebuda, 2016). Self-compassion’s negative relationship with neuroticism is well documented (Neff et al., 2007; Noack et al., 2025; Yang et al., 2023) and our findings extend this result. This result implies that individuals prone to negative affectivity may struggle to extend kindness toward themselves, which may impact creative self-beliefs.

Self-compassion had a small positive prediction of creative self-beliefs when alone; however, the predictive utility of self-compassion shifted once personality traits were introduced as this effect shifts to non-significant and negative (*β* = −0.06, *p* = 0.54). This suggests that initial predictive power of self-compassion is largely accounted for by shared variance with personality traits, most notably neuroticism. Based on VIF values between self-compassion and neuroticism at 1.79, this indicates that this is not an issue of collinearity, but rather, lack of unique variance between self-compassion and personality characteristics. Regression results indicate that self-compassion did not provide unique variance once neuroticism was controlled. This suggests that in this sample, the potential benefit of self-compassion was already explained by the absence of neuroticism. In other words, individuals low in Neuroticism may possess the emotional stability that self-compassion may promote. This extends previous findings on the relationship between self-compassion and neuroticism (Noack et al., 2025; Yang et al., 2023). As openness and conscientiousness were stronger contributors than self-compassion, this may suggest that when cognitive and motivational components associated with personality are considered, they are stronger than the emotional contribution of self-compassion.

Personality was a consistent factor in predicting creative self-beliefs, reflecting existing research linking personality to creative self-concept (Karwowski and Lebuda, 2017; Zandi et al., 2025). Openness was the strongest predictor of creative self-beliefs, which aligns with decades of research on the relationship between creativity and openness (Feist, 1998; Grajzel et al., 2023; Karwowski and Lebuda, 2016; Silvia et al., 2009). These findings reinforce that high openness promotes curiosity and imagination, as openness may support the development of creative self-beliefs (Pavlić et al., 2023). The positive contribution of Conscientiousness suggests that self-discipline and persistence may also enhance creative self-beliefs by promoting discipline, which allows ideas to turn into actions. Openness may promote idea generation and creativity, while conscientiousness may promote behavioral persistence. Our results reflect the connection between imaginative and effortful traits found in prior studies of creative self-beliefs (Karwowski and Lebuda, 2016).

Extraversion’s positive relationship with creative self-beliefs is consistent with existing literature (Jirásek and Sudzina, 2020; Puryear et al., 2017) reinforcing the behavioral and social components of the creative personality. Interestingly, extraversion was only a significant predictor of creative self-beliefs after categorical age was added to the model (*β* = 0.17, *p* < 0.05). Extraversion may play a stronger role during adolescence when social validation and peer feedback are particularly salient (Guyer et al., 2014). As individuals mature, creative self-beliefs may depend less on social pressures and more on internal cognitive and motivational factors, diminishing the importance of extraversion as a trait in favor of promoting openness and conscientiousness. The positive associations among extraversion and creative self-beliefs warrant further investigation into the specific social mechanisms that impact creative self-concept.

### 5.1 Limitations

While the results are promising, it is important to acknowledge the limitations of the current study. First, the sample primarily consisted of White participants from the Midwestern United States, which, although representative of the region, limits generalizability of findings to diverse populations. Future research should aim to include participants from more diverse geographic regions, racial backgrounds, ethnicities, sexual, and gender minorities. Second, our sample had vast age differences, and although our results indicated age was not a significant predictor, a more nuanced investigation of developmental stages may reveal otherwise. Future researchers are encouraged to investigate if there are developmental differences associated among self-compassion and creative self-beliefs with a larger sample. Third, all variables were assessed via self-report measures in a cross-sectional design, and common method bias could potentially impact results. While we acknowledge the inherent limitations of self-report data, results from the Harman’s Single-Factor Test indicated that the first factor accounted for only 19.10% of the total variance suggesting that measurement method was not a primary contributor to results. Nevertheless, future research should consider incorporating objective performance-based or behavioral measures of creativity or longitudinal designs to reduce these concerns. Fourth, this study only assessed main effects and future research using moderation analyses is required to determine if self-compassion functions as a buffer between neuroticism and creative self-beliefs.

### 5.2 Implications

Theoretically, these results refine the understanding of the creative personality by confirming established positive correlations between creative self-beliefs, extraversion, openness, and conscientiousness (Puryear et al., 2017). Our results suggest developmental differences specifically related to neuroticism and extraversion. While extraversion may be a driving factor for younger creatives, the role may shift based on internal experiences and mature reflection. It is possible that while younger creatives are negatively impacted by high neuroticism, they may develop emotional regulation strategies that do not inhibit creativity. This study contributes to the literature by exploring the shared variance between self-compassion and established personality predictors of creative self-beliefs.

Practically, these results have implications for education and counseling. Educational approaches may benefit from emphasizing activities promoting openness and conscientiousness as these traits consistently predicted creative self-beliefs and previous research demonstrated that curricula encouraging curiosity, persistence, and reflection may encourage students’ creativity (Beghetto et al., 2021). Classroom activities can move beyond divergent thinking exercises by emphasizing reflective practice and the methodical process of refining and editing, which are consistent with conscientiousness. Educational approaches that emphasize exploration, tolerance of ambiguity, and sustained effort may enhance creative confidence. Furthermore, counselors are encouraged to note the intricate effects of self-compassion on creative self-beliefs as correlations reveal a positive relationship. Counselors are encouraged to use interventions that promote self-kindness, common humanity, and mindfulness which are foundational to the Mindful Self-Compassion program (Neff and Germer, 2013). These exercises provide a non-trait-based avenue for increasing creative self-beliefs, which may be beneficial for creative individuals high in neuroticism. Since self-compassion can be learned and practiced through educational and therapeutic interventions this association suggests that self-compassion may be a promotive factor for creative self-beliefs, though its contribution overlaps with core personality traits. The malleable nature of self-compassion can be beneficial as interventions increasing self-compassion are possible. A practical issue for educators and counselors is that assessments of Big Five personality traits are expensive and usually limited in their use to licensed and certified professionals or researchers. Measures of creative self-beliefs and creative self-efficacy are less expensive, free, and not limited to their use to professional therapists and researchers. More investigation into self-compassion may provide a tangible and measurable psychological mechanism that may be used to enhance creative self-beliefs.

## 6 Conclusion

Ultimately, this study highlights that self-compassion predicts creative self-beliefs on its own; however, this relationship appears to contribute shared variance associated with personality traits. These findings suggest that while self-compassion may contribute to creative self-beliefs, these results are not only attributable to self-compassion. As this study was exploratory and aimed to assess relationships, more in-depth investigations with larger samples may reveal a more nuanced relationship. Self-compassion’s contribution appears to depend on the individual’s personality traits and potentially developmental stage, as evidenced by mean differences in personality traits. As openness and conscientiousness appeared to be the strongest determinants of creative self-beliefs, educational and counseling implications should strongly consider basing interventions on these factors. Our results suggest the necessity to continue investigating the relationships among creative self-beliefs, personality, and self-compassion across the lifespan and cultural contexts.

## Data Availability

The raw data supporting the conclusions of this article are not publicly available due to restrictions imposed by the University of Kansas Institutional Review Board (IRB) to protect participant privacy. Requests to access these data should be directed to the corresponding author, and access may be granted pending approval of a modification by the University of Kansas IRB.
